# Association between serum insulin levels and self-reported walking difficulty among adults with a history of stroke: A cross-sectional study of NHANES 1999–2018

**DOI:** 10.1097/MD.0000000000051480

**Published:** 2026-11-06

**Authors:** Tingting Fu, Anyin Wang, Jiang Peng, Xigui Tian, Xiaoqin Chen

**Affiliations:** a Department of Rehabilitation Medicine/Geriatrics, The People’s Hospital of Liangping District, Chongqing, China; b Department of Gastrointestinal/Hernia Surgery, The People’s Hospital of Liangping District, Chongqing, China; c Department of Internal Medicine-Neurology, The People’s Hospital of Liangping District, Chongqing, China; d Office of Hospital Administration, The People’s Hospital of Liangping District, Chongqing, China.

**Keywords:** cross-sectional study, NHANES, serum insulin, stroke, walking ability

## Abstract

Stroke is one of the leading causes of long-term disability worldwide. Walking difficulty is an important functional limitation among adults with a history of stroke. However, the association between serum insulin levels and self-reported walking difficulty in this population remains insufficiently studied. This study analyzed data from the National Health and Nutrition Examination Survey (NHANES) 1999–2018 to evaluate the association between serum insulin levels and self-reported walking difficulty among adults with a self-reported history of stroke. Weighted logistic regression models were used to adjust for confounders. Restricted cubic spline (RCS) analysis was conducted to explore potential nonlinear relationships. Sensitivity analyses were performed to assess the robustness of the findings. Subgroup analyses and interaction effect tests were conducted to examine heterogeneity across different populations. A total of 488 adults with a self-reported history of stroke were included in this study, representing 15,92,140 individuals in the US population. In the fully adjusted model, each 1 µU/mL increase in serum insulin level was associated with 5% higher odds of self-reported walking difficulty (OR = 1.05, 95% confidence interval: 1.01–1.09, *P* = .028). Quartile-based analysis showed that higher insulin levels (Q3, Q4) were significantly associated with greater odds of self-reported walking difficulty, with the highest quartile (Q4) showing an OR of 5.10 (95% confidence interval: 1.93–13.48, *P* = .001). RCS analysis suggested a possible nonlinear association between serum insulin levels and self-reported walking difficulty (*P* for non-linearity = .032). The associations observed in the sensitivity analyses were in the same direction as those in the primary analysis. Higher serum insulin levels were associated with greater odds of self-reported walking difficulty among adults with a self-reported history of stroke. These findings do not establish causality or a role for insulin in stroke-related motor recovery.

## 1. Introduction

Stroke is one of the leading causes of long-term disability worldwide.^[1]^ Many stroke survivors experience varying degrees of limb dysfunction, with walking impairment being an important functional limitation. Associated gait abnormalities and balance disorders can restrict mobility and daily activities, adversely affecting quality of life.^[2,3]^ Advances in acute stroke care have improved survival, but persistent disability and reduced independence remain major challenges.^[4,5]^ Notably, metabolic syndrome and its components have been investigated in relation to stroke risk.^[6,7]^

Studies have shown that insulin resistance (IR) is associated with poorer functional outcomes after ischemic stroke.^[8,9]^ A study conducted in Japan found that a high homeostasis model assessment of insulin resistance (HOMA-IR) value was independently associated with poor functional outcomes after acute ischemic stroke.^[10]^ In addition, diabetes **–** a common comorbidity of stroke **–** has been associated with alterations in cortical function and structure, which may be relevant to poststroke plasticity.^[11,12]^ Although previous studies have explored the relationship between IR and stroke severity or outcomes, the specific association between serum insulin levels and self-reported walking difficulty among adults with a history of stroke remains understudied. Population-based studies can complement evidence from acute stroke cohorts by examining walking difficulty among adults with a history of stroke in community settings. Such analyses should account for potential confounding factors. These include age, sex, race, comorbidities (e.g., diabetes, hypertension), lifestyle factors (e.g., smoking, alcohol consumption), and socioeconomic status. These variables may be related to both serum insulin levels and walking difficulty, potentially confounding the observed association.

Using data from the National Health and Nutrition Examination Survey (NHANES) 1999–2018, this study examined the association between serum insulin levels and self-reported walking difficulty among adults with a self-reported history of stroke. Weighted multivariable logistic regression was used to adjust for measured confounders, and restricted cubic spline (RCS) analysis explored potential nonlinearity.

## 2. Materials and methods

### 2.1. Data source and study population

This study utilized publicly available data from the NHANES spanning 1999–2018. NHANES is a nationally representative cross-sectional survey conducted by the Centers for Disease Control and Prevention to assess the health and nutritional status of adults and children in the United States. The survey employs a complex, stratified, multistage probability sampling design to ensure national representativeness. NHANES data include demographic information, clinical examination results, laboratory test findings, and health-related questionnaires, covering a wide range of health indicators and disease-related variables. The data collection process follows strict quality control standards. The NHANES data used in this study were de-identified and publicly available. According to the Declaration of Helsinki and relevant ethical guidelines, studies using publicly available NHANES data do not require additional ethical approval or informed consent. Therefore, this study did not seek separate institutional ethical approval or obtain additional participant consent.^[13]^

This study analyzed data from NHANES 1999–2018, including all individuals who participated in the survey during this period. Participants were excluded if they were under 20 years of age, had no history of stroke or missing stroke history data, lacked information on self-reported walking difficulty, or had missing serum insulin level data.

### 2.2. Assessment of self-reported stroke history

A history of stroke was identified from an affirmative response to the NHANES questionnaire variable MCQ160f (“Has a doctor or other health professional ever told you that you had a stroke?”). This variable applies to adults aged 20 years and older. Stroke history was based on participants’ reports of a diagnosis by a doctor or other health professional and was not independently verified using medical records or neuroimaging.

### 2.3. Assessment of self-reported walking difficulty

Self-reported walking difficulty was assessed using NHANES questionnaire item PFQ061B, which asks about difficulty walking approximately one-quarter mile (2 to 3 blocks) independently and without special equipment. Respondents were instructed not to consider temporary conditions, such as pregnancy or broken limbs. Responses were categorized as no walking difficulty (code 1, “No difficulty”) or walking difficulty (codes 2–4, indicating “Some difficulty,” “Much difficulty,” or “Unable to do”). This item assesses general walking difficulty and does not establish whether the limitation is attributable to stroke.

### 2.4. Measurement of serum insulin

Serum insulin levels were measured by NHANES contract laboratories according to standardized laboratory protocols. Insulin measurements were available in all ten 2-year survey cycles from 1999–2000 through 2017–2018. Serum samples for insulin measurement were collected from eligible participants aged 12 years and older during the morning mobile examination center (MEC) session; therefore, insulin measurements were not available for the entire examined sample.

The analytical methods varied across survey cycles. Serum insulin was measured using a Pharmacia double-antibody radioimmunoassay in 1999 to 2002, a Tosoh AIA-PACK IRI 2-site immunoenzymometric assay in 2003 to 2004, and a Mercodia 2-site sandwich enzyme-linked immunosorbent assay in 2005 to 2008. During 2009 to 2010, the method changed from the Mercodia assay to a Roche Elecsys 2010 chemiluminescent immunoassay in late 2009. NHANES adjusted the 2010 insulin results to improve comparability with the 2009 results. The Roche assay was used in 2011 to 2012, and the Tosoh AIA-PACK IRI assay was used in 2013 to 2018. Testing was performed at the University of Missouri–Columbia in 1999 to 2004 and 2013 to 2018 and at the University of Minnesota in 2005 to 2012.

Laboratory quality assurance and quality control followed NHANES protocols, with detailed specimen handling and analytical procedures provided in the corresponding cycle-specific laboratory documentation. The measured serum insulin concentrations were recorded in units of μU/mL. Serum insulin values were used as provided in the publicly released NHANES datasets, without additional cross-cycle assay calibration.

### 2.5. Covariates

To control for potential confounding factors, the following covariates were included in the analysis: age, sex, race/ethnicity (Mexican American/Other Hispanic, Non-Hispanic White, Non-Hispanic Black/Other race), education level (less than high school, high school or equivalent, college or higher),^[14]^ marital status (married/living with a partner, widowed/divorced/separated/never married), poverty-to-income ratio (PIR), body mass index (BMI; <25 kg/m^2^, ≥25 kg/m^2^),^[15]^ smoking status (never smoker, former smoker, current smoker),^[16]^ daily alcohol intake (0 g/d, >0 g/d), diabetes (yes/no), hypertension (yes/no), total cholesterol, triglycerides, serum creatinine, white blood cell count, fasting glucose, and glycated hemoglobin (HbA1c). For certain categorical variables, such as race/ethnicity and marital status, categories with small sample sizes were merged with adjacent groups to ensure statistical model stability and reduce bias.

### 2.6. Statistical analyses

The complex sampling design of NHANES was accounted for by incorporating fasting subsample weights, strata (SDMVSTRA), and primary sampling units (SDMVPSU). Serum insulin levels were analyzed as both a continuous variable and quartiles (Q1–Q4), while self-reported walking difficulty was treated as a binary outcome (yes/no). Baseline characteristics were summarized using mean ± standard deviation for normally distributed continuous variables and median (interquartile range) for non-normally distributed data, with analysis of variance or the Kruskal–Wallis test used for group comparisons. Categorical variables were presented as frequency (percentage), and comparisons were conducted using the chi-square (χ^2^) test.

In the association analysis, missing values were first excluded, and then weighted logistic regression models were used to evaluate the preliminary relationship between serum insulin levels and self-reported walking difficulty. The results were presented as odds ratios (ORs) with 95% confidence intervals (CIs). Three weighted logistic regression models were fitted: model 1 was unadjusted; model 2 adjusted for sex, age, education level, marital status, race, and PIR; and model 3 further adjusted for BMI, smoking status, daily alcohol intake, diabetes, hypertension, total cholesterol, triglycerides, serum creatinine, white blood cell count, fasting glucose, and HbA1c. To assess potential nonlinear relationships, RCS analysis was used to examine potential nonlinearity in the association between serum insulin levels and self-reported walking difficulty. Additionally, subgroup analyses and interaction effect tests were conducted. Two sensitivity analyses were performed: excluding participants with serum insulin levels in the top and bottom 1% and excluding diabetic patients with HbA1c ≥ 7%. All statistical analyses were conducted using R software version 3.3.2 (R Foundation for Statistical Computing) and Free Statistics software version 1.9 (Beijing FreeClinical Medical Technology Co., Ltd.), with a 2-sided significance level of α = 0.05. *P*-values < .05 were considered statistically significant.

## 3. Results

### 3.1. Selection of participants

This study utilized data from the 1999–2018 NHANES database, which initially included 101,316 participants. First, individuals under 20 years of age (n = 46,235) were excluded, leaving 55,081 participants. Next, participants without a history of stroke or with missing stroke status data (n = 52,884) were further excluded, resulting in 2197 participants with a self-reported history of stroke. Among them, individuals with missing self-reported walking difficulty data (n = 1092) were excluded, leaving 1105 participants. Finally, after excluding individuals with missing serum insulin data (n = 617), a total of 488 participants were included in the final analysis, as shown in Figure 1.

**Figure 1.**
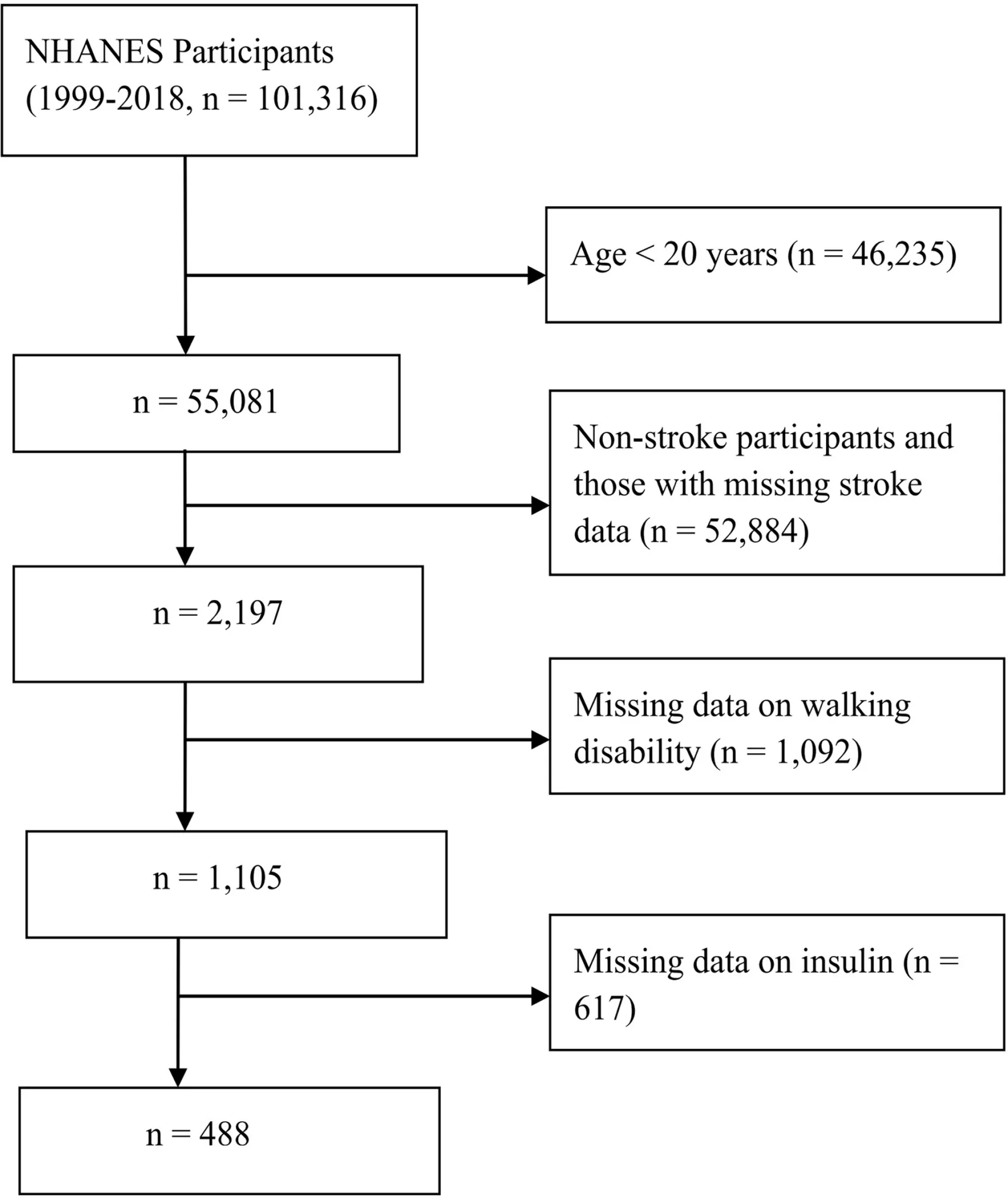
Flowchart outlining the study design and participant selection process. NHANES = National Health and Nutrition Examination Survey.

### 3.2. Baseline characteristics of study participants

A total of 488 adults with a self-reported history of stroke were included in the final analysis, representing a weighted population of 1,592,140 individuals in the United States. Among the 488 participants, 269 (55.1%) reported no walking difficulty, whereas 219 (44.9%) reported walking difficulty. The mean age was 68.7 ± 11.4 years in the participants without self-reported walking difficulty and 68.0 ± 12.0 years in the participants with self-reported walking difficulty. Significant differences were observed between the 2 groups in marital status, PIR, diabetes prevalence, daily alcohol intake, and white blood cell count, while no statistically significant differences were found for other variables (Table 1).

**Table 1:** Baseline characteristics of the study participants.

Variables	Total (n = 488)	Without walking difficulty (n = 269)	With walking difficulty (n = 219)	*P*-value
Weighted sample size	1,592,140	927,602	664,538	
Age (yr), Mean ± SD	68.4 ± 11.7	68.7 ± 11.4	68.0 ± 12.0	.993
Sex, n (%)				.159
Male	246 (50.4)	149 (55.4)	97 (44.3)	
Female	242 (49.6)	120 (44.6)	122 (55.7)	
Race/ethnicity, n (%)				.449
Mexican American/Other Hispanic	72 (14.8)	46 (17.1)	26 (11.9)	
Non-Hispanic White	267 (54.7)	139 (51.7)	128 (58.4)	
Non-Hispanic Black/Other Race	149 (30.5)	84 (31.2)	65 (29.7)	
Education, n (%)				.212
Less than high school	177 (36.3)	83 (30.9)	94 (42.9)	
High school or equivalent	128 (26.2)	73 (27.1)	55 (25.1)	
College or above	183 (37.5)	113 (42)	70 (32)	
Marital status, n (%)				.033
Married/living with partner	285 (58.4)	169 (62.8)	116 (53)	
Widowed/divorced/separated/never married	197 (40.4)	98 (36.4)	99 (45.2)	
Missing data	6 (1.2)	2 (0.7)	4 (1.8)	
PIR, Median (IQR)	1.7 (1.1, 3.1)	2.0 (1.1, 3.5)	1.4 (1.0, 2.4)	<.001
Missing data, n (%)	38 (7.8)	18 (3.7)	20 (4.1)	
BMI, n (%)				.746
<25 kg/m^2^	132 (27.0)	86 (32)	46 (21)	
≥25 kg/m^2^	346 (70.9)	181 (67.3)	165 (75.3)	
Missing data	10 (2.0)	2 (0.7)	8 (3.7)	
Smoking status, n (%)				.531
Never	192 (39.3)	111 (41.3)	81 (37)	
Former	190 (38.9)	106 (39.4)	84 (38.4)	
Current	106 (21.7)	52 (19.3)	54 (24.7)	
Daily alcohol intake, n (%)				.038
0 g/d	397 (81.4)	209 (77.7)	188 (85.8)	
> 0 g/d	65 (13.3)	43 (16)	22 (10)	
Missing data	26 (5.3)	17 (6.3)	9 (4.1)	
Diabetes, n (%)				.029
No	356 (73.0)	201 (74.7)	155 (70.8)	
Yes	132 (27.0)	68 (25.3)	64 (29.2)	
Hypertension, n (%)				.074
No	90 (18.4)	58 (21.6)	32 (14.6)	
Yes	393 (80.5)	209 (77.7)	184 (84)	
Missing data	5 (1.0)	2 (0.7)	3 (1.4)	
Total cholesterol (mg/L), Mean ± SD	190.6 ± 47.2	187.6 ± 47.2	194.4 ± 47.0	.767
Missing data, n (%)	2 (0.4)	1 (0.2)	1 (0.2)	
Triglycerides (mg/dL), Median (IQR)	115.0 (79.0, 172.0)	113.0 (79.0, 158.0)	129.5 (79.0, 185.0)	.098
Missing data, n (%)	7 (1.4)	4 (0.8)	3 (0.6)	
Serum creatinine (mg/dL), Median (IQR)	1.0 (0.8, 1.2)	1.0 (0.8, 1.2)	1.0 (0.8, 1.2)	.714
Missing data, n (%)	3 (0.6)	2 (0.4)	1 (0.2)	
White blood cell (×10^9^/L), Mean ± SD	7.1 ± 2.5	6.8 ± 1.8	7.5 ± 3.2	.011
Missing data, n (%)	2 (0.4)	1 (0.2)	1 (0.2)	
Fasting blood glucose (mg/dL), Mean ± SD	117.9 ± 42.7	118.0 ± 42.8	117.7 ± 42.6	.079
HbA1c (%), Mean ± SD	6.0 ± 1.2	6.1 ± 1.3	6.0 ± 1.1	.830

BMI = body mass index, HbA1c = glycated hemoglobin, IQR = interquartile range, PIR = family income-to-poverty ratio, SD = standard deviation.

### 3.3. Association between serum insulin levels and self-reported walking difficulty among adults with a history of stroke

In the fully adjusted model 3, each 1 µU/mL increase in serum insulin levels was associated with 5% higher odds of self-reported walking difficulty (OR = 1.05, 95% CI: 1.01–1.09, *P* = .028). Compared with Q1, the ORs were 1.30 (95% CI: 0.54–3.13, *P* = .548) for Q2, 2.77 (95% CI: 1.10–6.97, *P* = .031) for Q3, and 5.10 (95% CI: 1.93–13.48, *P* = .001) for Q4 (*P* for trend < .001; Table 2).

**Table 2:** Association between serum insulin levels and self-reported walking difficulty in different logistic regression models.

	Model 1		Model 2		Model 3	
	OR (95% CI)	*P*-value	OR (95% CI)	*P*-value	OR (95% CI)	*P*-value
Continuous	1.04 (1.01, 1.07)	.004	1.05 (1.02, 1.08)	.004	1.05 (1.01, 1.09)	.028
Quartile						
Q1 (1.06–6.48 µU/mL, n = 122)	Reference		Reference		Reference	
Q2 (6.51–10.64 µU/mL, n = 122)	1.26 (0.53, 2.97)	.599	1.36 (0.64, 2.88)	.423	1.30 (0.54, 3.13)	.548
Q3 (10.78–17.39 µU/mL, n = 122)	2.15 (0.96, 4.79)	.062	2.47 (1.15, 5.29)	.021	2.77 (1.10, 6.97)	.031
Q4 (17.44–255.46 µU/mL, n = 122)	3.86 (1.65, 9.01)	.002	4.55 (1.93, 10.71)	<.001	5.10 (1.93, 13.48)	.001
*P* for trend		<.001		<.001		<.001

Model 1: No covariates included.

Model 2: Adjusted for demographic factors: age, sex, race/ethnicity, education level, marital status, and PIR.

Model 3: Additionally adjusted for BMI, smoking status, daily alcohol intake, diabetes, hypertension, total cholesterol, triglycerides, serum creatinine, white blood cell count, fasting glucose, and HbA1c.

BMI = body mass index, CI = confidence interval, HbA1c = glycated hemoglobin, OR = odds ratio, PIR = poverty-to-income ratio.

### 3.4. Results of RCS analysis

An RCS regression model was used to explore the dose-response relationship between serum insulin levels and self-reported walking difficulty among adults with a history of stroke. The results indicated a significant nonlinear relationship (*P* for non-linearity = .032) and an overall statistically significant trend (*P* for overall < .001), as shown in Figure 2.

**Figure 2.**
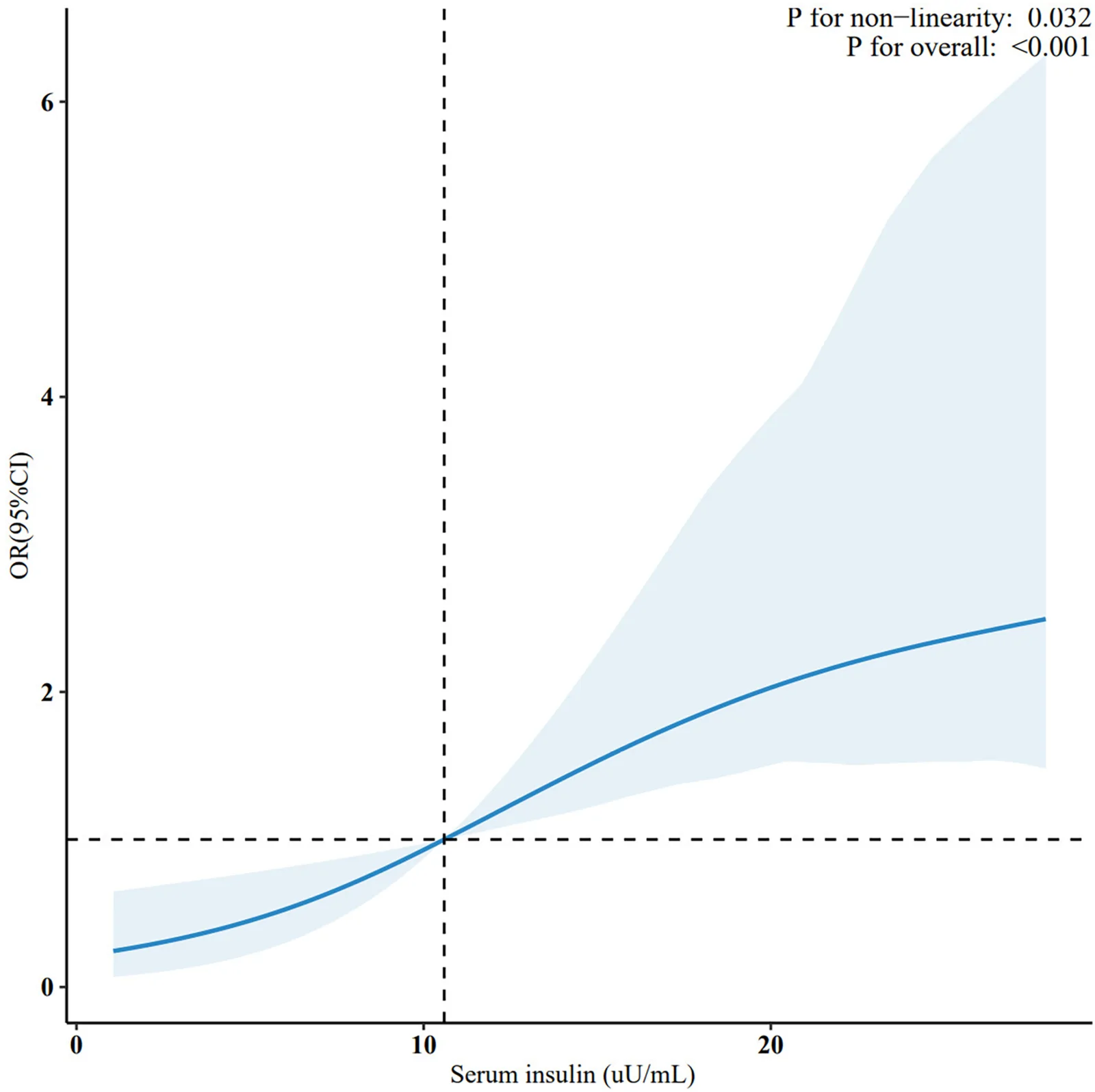
RCS illustrating the multivariable-adjusted relationship between serum insulin levels and self-reported walking difficulty among adults with a history of stroke. CI = confidence interval, OR = odds ratio, RCS = restricted cubic spline.

### 3.5. Results of sensitivity analyses

After excluding participants with serum insulin levels in the upper or lower 1%, the fully adjusted OR per 1 µU/mL increase was 1.07 (95% CI: 1.03–1.11, *P* = .001; Table 3). After excluding participants with diabetes and HbA1c ≥ 7%, the corresponding OR was 1.05 (95% CI: 1.00–1.09, *P* = .049; Table 4). Both estimates were in the same direction as the primary estimate.

**Table 3:** Sensitivity analysis: association between serum insulin levels and self-reported walking difficulty after excluding participants in the top and bottom 1% of serum insulin levels.

	Model 1		Model 2		Model 3	
	OR (95% CI)	P-value	OR (95% CI)	*P*-value	OR (95% CI)	*P*-value
Continuous	1.05 (1.02, 1.08)	<.001	1.06 (1.03, 1.09)	<.001	1.07 (1.03, 1.11)	.001
Quartile						
Q1 (2.14–6.59 µU/mL, n = 120)	Reference		Reference		Reference	
Q2 (6.62–10.64 µU/mL, n = 119)	1.40 (0.58, 3.35)	.445	1.48 (0.70, 3.12)	.299	1.51 (0.62, 3.64)	.357
Q3 (10.78–16.92 µU/mL, n = 118)	2.35 (1.03, 5.34)	.041	2.72 (1.28, 5.78)	.010	3.16 (1.24, 8.05)	.017
Q4 (17.31–91.88 µU/mL, n = 121)	4.11 (1.75, 9.69)	.002	5.14 (2.23, 11.86)	<.001	5.81 (2.22, 15.19)	<.001
*P* for trend		<.001		<.001		<.001

Model 1: No covariates included.

Model 2: Adjusted for demographic factors: age, sex, race/ethnicity, education level, marital status, and PIR.

Model 3: Additionally adjusted for BMI, smoking status, daily alcohol intake, diabetes, hypertension, total cholesterol, triglycerides, serum creatinine, white blood cell count, fasting glucose, and HbA1c.

BMI = body mass index, CI = confidence interval, HbA1c = glycated hemoglobin, OR = odds ratio, PIR = poverty-to-income ratio.

**Table 4:** Sensitivity analysis: association between serum insulin levels and self-reported walking difficulty after excluding diabetic patients with HbA1c ≥ 7%.

	Model 1		Model 2		Model 3	
	OR (95% CI)	*P*-value	OR (95% CI)	*P*-value	OR (95% CI)	*P*-value
Continuous	1.04 (1.01, 1.07)	.023	1.04 (1.01, 1.07)	.022	1.05 (1.00, 1.09)	.049
Quartile						
Q1 (1.06–6.31 µU/mL, n = 108)	Reference		Reference		Reference	
Q2 (6.40–10.19 µU/mL, n = 109)	1.16 (0.51, 2.64)	.719	1.42 (0.67, 2.98)	.351	1.47 (0.65, 3.34)	.350
Q3 (10.36–16.16 µU/mL, n = 107)	1.89 (0.83, 4.30)	.130	2.24 (1.00, 5.01)	.05	2.72 (1.02, 7.30)	.047
Q4 (16.35–141.27 µU/mL, n = 108)	3.08 (1.27, 7.48)	.014	3.25 (1.30, 8.14)	.013	4.83 (1.65, 14.15)	.005
*P* for trend		.006		.005		.003

Model 1: No covariates included.

Model 2: Adjusted for demographic factors: age, sex, race/ethnicity, education level, marital status, and PIR.

Model 3: Additionally adjusted for BMI, smoking status, daily alcohol intake, diabetes, hypertension, total cholesterol, triglycerides, serum creatinine, white blood cell count, fasting glucose, and HbA1c.

BMI = body mass index, CI = confidence interval, HbA1c = glycated hemoglobin, OR = odds ratio, PIR = poverty-to-income ratio.

### 3.6. Results of Subgroup and Interaction Effect Analyses

Subgroup analyses were conducted to explore the association between serum insulin levels and self-reported walking difficulty across subgroups of adults with a history of stroke. The subgroup analysis results showed nominally significant associations (*P* < .05, without adjustment for multiple comparisons) within the following groups: females (OR = 1.07, 95% CI: 1.02–1.12, *P* = .01), individuals with higher education (College or above, OR = 1.14, 95% CI: 1.05–1.25, *P* = .01), those who are married or living with a partner (OR = 1.11, 95% CI: 1.03–1.18, *P* = .01), individuals with a BMI < 25 kg/m^2^ (OR = 1.19, 95% CI: 1.05–1.35, *P* < .01), never-smokers (OR = 1.04, 95% CI: 1.01–1.07, *P* = .01), former smokers (OR = 1.06, 95% CI: 1.02–1.10, *P* < .01), those with a daily alcohol intake of 0 g/d (OR = 1.04, 95% CI: 1.00–1.09, *P* = .04), and patients with hypertension (OR = 1.06, 95% CI: 1.02–1.11, *P* < .01).

The interaction test for marital status yielded a nominal *P*-value of .03. No statistically significant interactions were found for other variables (*P* > .05). Figure 3 presents detailed results.

**Figure 3.**
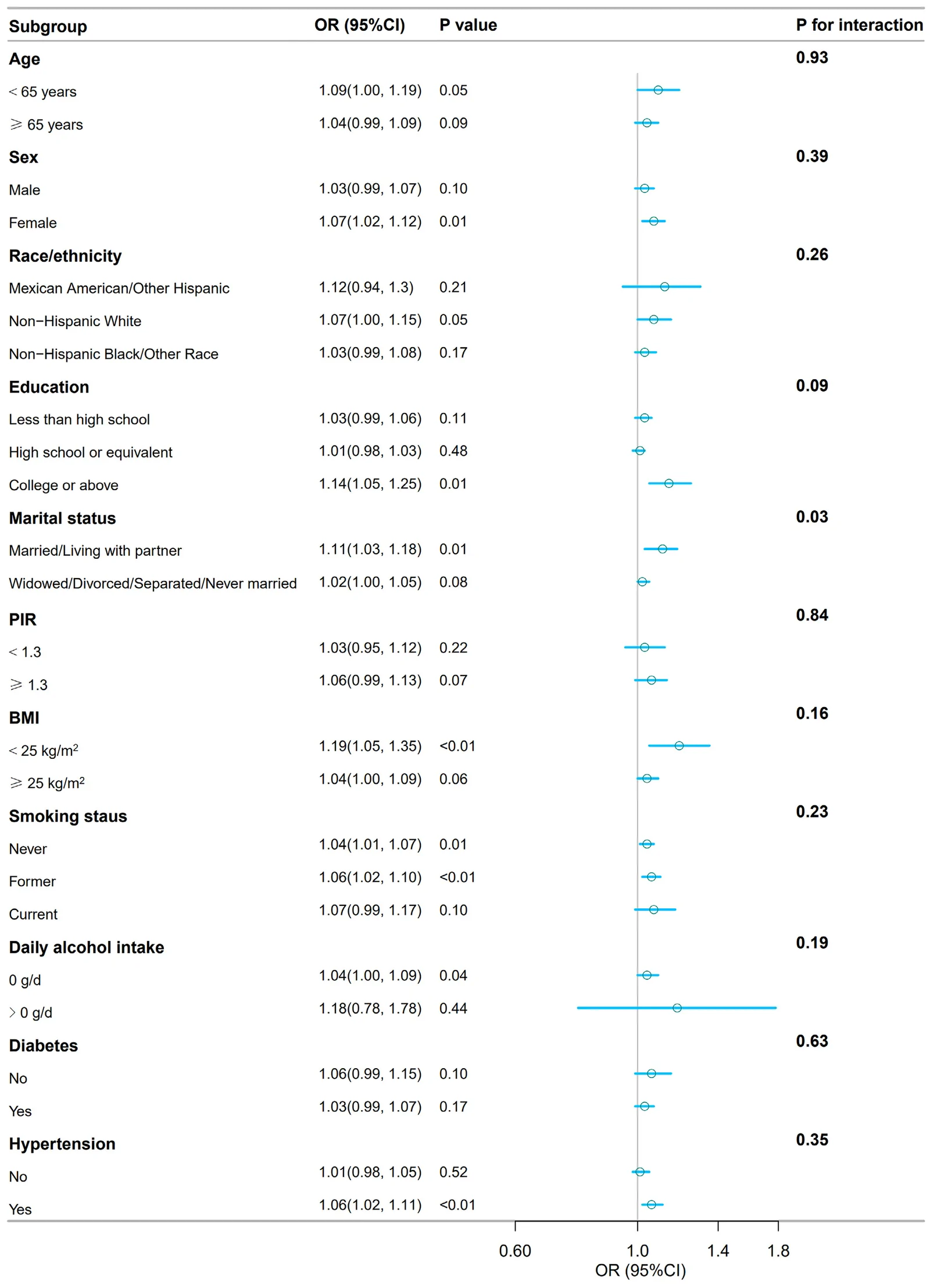
Results of subgroup analyses and interaction effect evaluations. CI = confidence interval, OR = odds ratio.

## 4. Discussion

In this cross-sectional analysis of NHANES 1999–2018 data, higher serum insulin levels were associated with greater odds of self-reported walking difficulty among adults with a self-reported history of stroke after adjustment for measured covariates. Exploratory RCS analysis suggested a possible nonlinear association, and estimates from the sensitivity analyses were in the same direction as the primary estimate.

Previous studies have examined associations of IR and circulating insulin levels with stroke-related outcomes, although these represent distinct metabolic measures. Chang et al assessed IR scores to evaluate the severity of IR and used multivariable logistic regression analysis, adjusting for age and sex, to examine its relationship with functional prognosis. Their findings indicated that IR was associated with poor functional outcomes in nondiabetic patients with acute ischemic stroke.^[17]^ In a mouse model study, Vercalsteren et al investigated the impact of IR on poststroke functional recovery. The results showed that prestroke induction of IR worsened neurological recovery, whereas normalizing IR before stroke improved recovery. These findings suggest that future clinical research should focus on prestroke IR to reduce poststroke complications in diabetic and prediabetic elderly patients.^[18]^ Lakka et al measured fasting serum insulin levels and conducted a 9.5-year follow-up to assess the association between hyperinsulinemia and acute coronary/cerebrovascular events (including stroke) in men. Their results demonstrated a direct relationship between hyperinsulinemia and stroke risk (*P* = .02). However, the association was attenuated after adjustment for lipid levels, blood pressure, and obesity. These findings highlight the importance of comprehensive metabolic risk factor management in stroke prevention.^[19]^

This study has several strengths: it is based on NHANES, a nationally representative survey with standardized data collection methods. A multivariable weighted logistic regression model was used to adjust for measured potential confounders, including age, sex, comorbidities (e.g., diabetes, hypertension), lifestyle factors (e.g., smoking, alcohol consumption), and socioeconomic status. RCS analysis suggested a possible nonlinear association between serum insulin levels and self-reported walking difficulty among adults with a history of stroke. Sensitivity analyses assessed the stability of the estimates under alternative exclusion criteria, while exploratory subgroup and interaction analyses examined potential heterogeneity. These findings add population-based evidence on the association between serum insulin levels and self-reported walking difficulty among adults with a history of stroke and provide a basis for further investigation.

Previous studies suggest that IR may influence poststroke recovery through several potential mechanisms: IR promotes neuroinflammatory responses, affecting functional recovery after stroke.^[18,20]^ It increases the release of pro-inflammatory cytokines, such as tumor necrosis factor-α and interleukin-6.^[21,22]^ Tumor necrosis factor-α and interleukin-6 were not assessed in the present analysis. White blood cell counts were higher among participants with self-reported walking difficulty, but this nonspecific finding cannot establish neuroinflammation or impaired neural repair. Additionally, under IR, ischemic stroke recovery is influenced by both oxidative stress and neuroinflammation, further worsening poststroke prognosis.^[23,24]^ IR disrupts the 5′ AMP-activated protein kinase signaling pathway, reducing energy supply to brain tissue and impairing poststroke neuroregeneration.^[25–27]^ Persistent IR leads to downregulation of brain insulin receptors, further exacerbating poststroke neurological impairment.^[20,28]^ IR impairs muscle glycogen synthesis, reducing the effectiveness of rehabilitation training.^[29,30]^ This metabolic disruption affects walking ability and daily living activities in stroke patients.^[31]^ These mechanisms were not evaluated in the present study, and their relevance to the observed association remains uncertain.

Interventions targeting insulin sensitivity have been investigated in stroke research. Potential neuroprotective effects of metformin have been discussed in previous reviews.^[25]^ In a small preliminary study of people with chronic stroke, resistance training reduced fasting insulin, improved insulin sensitivity, and improved lower-limb strength.^[32]^ An observational study reported an association between higher HOMA-IR and greater ischemic stroke severity in patients without diabetes.^[33]^ However, the present study cannot determine whether interventions targeting insulin sensitivity improve walking outcomes.

This study has several limitations. First, as a cross-sectional study, it can only reveal an association between serum insulin levels and self-reported walking difficulty among adults with a history of stroke but cannot establish causality. Self-reported walking difficulty was assessed during the household interview, whereas serum insulin was measured in blood samples collected during the subsequent mobile examination center visit within the same survey cycle. This sequence of data collection does not establish whether elevated serum insulin preceded the onset of walking difficulty, and reverse causation cannot be excluded. Second, stroke history was self-reported and was not verified against medical records or neuroimaging, which may have resulted in recall bias and misclassification. Stroke subtype (ischemic vs hemorrhagic), severity, and lesion location were not characterized, and time since stroke was not evaluated in the present analysis. Consequently, we could not assess whether the observed association differed according to these clinical characteristics, and residual confounding by these factors may remain. Third, walking difficulty was assessed using a single self-reported questionnaire item, without objective gait or walking endurance assessments. This measure cannot distinguish stroke-related impairment from walking limitations due to other conditions or establish whether the difficulty began after the stroke. Therefore, the findings should be interpreted as an association with general walking difficulty among adults with a history of stroke, rather than evidence concerning stroke-specific neurological impairment or recovery. Fourth, although multiple confounding factors were adjusted for, residual confounding may still exist. Factors such as individual rehabilitation training were not accounted for, which may have confounded the observed association. Fifth, this study included only participants with a history of stroke and complete serum insulin data, which may have introduced selection bias and limited the generalizability of the findings. Sixth, IR was not directly measured, and no IR index was included in the analysis. Serum insulin concentration alone does not establish IR; therefore, the observed association cannot be attributed specifically to IR. Differences in insulin assays across survey cycles may have introduced measurement heterogeneity, as no additional cross-cycle assay calibration was performed in this analysis. Finally, multiple analyses were performed without adjustment for multiple comparisons, increasing the possibility of false-positive findings. The subgroup, interaction, and spline results should therefore be interpreted as exploratory, and the sensitivity analyses should not be regarded as independent confirmation of the findings.

## 5. Conclusion

This study identified an association between higher serum insulin levels and greater odds of self-reported walking difficulty among adults with a self-reported history of stroke using data from the 1999–2018 NHANES database. These findings do not establish causality or a role for insulin in stroke-related motor recovery. Prospective studies incorporating objective walking assessments and detailed stroke characteristics are needed to clarify this association.

## Acknowledgments

We thank DeepL Translator for its assistance in language refinement and improving the readability of the text. Additionally, all authors independently reviewed the manuscript to ensure that the original meaning was not altered during the editing process.

## Author contributions

**Conceptualization:** Tingting Fu, Anyin Wang, Xiaoqin Chen.

**Data curation:** Tingting Fu, Jiang Peng, Xiaoqin Chen.

**Formal analysis:** Tingting Fu, Jiang Peng.

**Investigation:** Xigui Tian.

**Methodology:** Tingting Fu, Anyin Wang.

**Software:** Tingting Fu, Anyin Wang.

**Supervision:** Anyin Wang.

**Validation:** Jiang Peng, Xigui Tian.

**Visualization:** Tingting Fu, Anyin Wang.

**Writing – original draft:** Tingting Fu, Anyin Wang, Jiang Peng, Xigui Tian, Xiaoqin Chen.

**Writing – review & editing:** Tingting Fu, Anyin Wang, Jiang Peng, Xigui Tian, Xiaoqin Chen.
